# Training and match load ratios in professional soccer–should we use player- or position-specific match reference values?

**DOI:** 10.3389/fspor.2023.1151828

**Published:** 2023-05-16

**Authors:** Linda Ammann, Stefan Altmann

**Affiliations:** ^1^ Integrative and Experimental Exercise Science, Department of Sport Science, University of Würzburg, Würzburg, Germany; ^2^ Institute of Sports and Sports Science, Karlsruhe Institute of Technology, Karlsruhe, Germany; ^3^ TSG ResearchLab gGmbH, Zuzenhausen, Germany

**Keywords:** load management, soccer, training, match, physical performance, team sports

## Abstract

Careful load management is needed to optimize the physical capacity, a key performance component, of soccer players. The training load of soccer players is often expressed as a percentage of match load. However, no study has yet evaluated how training match load ratios are affected by using either a player-specific or position-specific reference for match load. Therefore, this study aimed to compare training match load ratios of professional soccer players per day of a microcycle with match load being player-specific and position-specific, respectively. Additionally, the load that players typically experience per day of a microcycle and its variation should be analyzed. Therefore, a retrospective observational cohort study was conducted over a 14-month period, analyzing 11 external load measures during sessions of 20 players belonging to a team competing in the highest Swiss league. Within a microcycle, typical full matches presented a unique load for players, and they experienced higher training loads on days with a greater temporal distance to a match. Load variation proved to be highly associated with the day in a microcycle and the load measure. Substantial differences in typical load were evident in (i) trainings between players, (ii) matches both between players and positions, and (iii) training match load ratios when using player-specific or position-specific match references. The importance of individual load management in professional soccer was reaffirmed. When consulting training match load ratios for that purpose, one should be aware of the aim, select appropriate reference values depending on it, and interpret the ratios accurately to finally draw adequate conclusions.

## Introduction

The performance of soccer players depends on different components, among which, physical, technical, and tactical performance, as well as psychological factors, are the most important (1–4). Therefore, soccer players should aim to optimize their physical capabilities in both the short and long term. Training and competition load can be defined as an input variable that is usually tried to be manipulated when intending to elicit certain training induced adaptations (as this term including competitions) (5).

Knowing what soccer players physically need to be prepared for requires a solid understanding of the sport-specific demands (6–9). Measures of load, both in training and matches, can be categorized as either external or internal, depending on whether they refer to measurable aspects occurring externally or internally to the athlete (10–12). External loads are objective measures of the work performed by an athlete. In contrast, internal loads refer to the relative biological (both physiological and psychological) stressors imposed on the athlete (10, 11). Nowadays, there are a number of methods for measuring training and match loads (10, 12–14).

In order to optimally develop the physical capabilities of soccer players, training stimuli need to be applied individually even within a team environment (10, 13–16). To individually tailor training programs, maximizing positive physiological adaptation and simultaneously preventing injury and illness, careful load monitoring is required (10–13, 17). Within load monitoring, employing an integrated load monitoring approach (i.e., rigorous and consistent, combining both external and internal loads) seems crucial (10–13, 15).

The training load of soccer players in a session or, if multiple sessions are undertaken per day, in a day of a microcycle (i.e., periods of training lasting from the first to the last day focused on a match, and whose length may vary depending on the competitive calendar) can be expressed in relation to match load (18–20). To the best of the authors' knowledge, this is done in the field more often than one might expect based on the available body of literature. Comparable limited scientific investigations may be mainly since training and match load are still often measured with different tracking systems, not allowing for direct comparisons in scientific research (12). It is evident that match load varies according to playing position (2, 7–9, 21, 22); however, limited data exist presenting the in-season training loads of professional soccer players per day of a microcycle and relative to match load across positions (18) or allowing (23) such ratios to be built across positions (e.g., missing or incomplete data, microcycle accumulated load, averaged load). Recently, data from Altmann et al. (21) showed that physical match performance does not only depend on the playing position but also, to a considerable extent, on the individual player, irrespective of playing position. Thus, the question arises of whether position-specific or player-specific reference values should be used when building training match load ratios. To the best of our knowledge, no study has yet been conducted to address this question.

Furthermore, loading patterns observed in one team are of limited generalizability, as match demands vary depending on tactical formation or contextual factors (e.g., match location, environmental conditions, match importance, preparation, fixture congestion, season phase, match outcome, nutrition strategies, and game rules) (2–4, 7–9, 21, 24–27), and training practices may differ depending on coaches’ training philosophies or situational conditions (18, 19, 23). Thus, findings from further populations may contribute to a more complex understanding of current practices.

Therefore, in the pursuit of sound practices, the primary aim of this study is to determine and compare the training-match load ratios of professional soccer players belonging to a team competing in the highest Swiss league (Credit Suisse Super League®) per day of a microcycle, with match load being player-specific and position-specific, respectively. To get a more complete picture, first, the load that players experience per day of a microcycle should be analyzed in absolute numbers. In doing so, it should be quantified and its variation assessed.

## Materials and methods

### Participants

49 from a total of *N* = 53 elite male professional soccer field players participating in training and/or match sessions for the first team of a Swiss club were asked to take part in this study. All of them gave written informed consent voluntarily. Goalkeepers were excluded due to their different activity profile compared to field positions (28), and four field players were not asked to participate since they left the club before the researchers had the opportunity to invite the eligible players. All players were screened for health contraindications by the internal club sports medicine staff as part of their normal care of the team. As all data used in the current study arose from routine monitoring, no ethical approval was required (29). Players with <5 in-season sessions were excluded from analysis (*n* = 11), as they were not part of the team for at least one in-season microcycle. To be able to compare player-specific and position-specific data, from the remaining players, those who did not have a minimum of three individual match observations fulfilling the criteria outlined below were also excluded (*n* = 18). A lower limit of three matches was set to reduce the effects of possible match-to-match variation in the external load (2–4, 7, 21, 24–27). Thus, the sample size considered for the analysis was *n* = 20.

### Study design and research methods

A retrospective observational cohort study was implemented over the course of 14 months, starting during the first half of the 2021/22 season and lasting until the end of the first half of the 2022/23 season. In this phase, the team under observation competed in the highest-level national championship (Credit Suisse Super League®) as well as the national cup competition (Helvetia Schweizer Cup) in Switzerland. In addition to 48 championship matches and 6 cup matches, there were 14 test matches.

The data analyzed were derived from daily routine monitoring of the players. Data from rehabilitation, strength, or additional off-court recreational training sessions were excluded. The same applies to on-field sessions which a player completed outside the team mentioned (e.g., U21 team). Training sessions included all the activities on the pitch (i.e., warm-up, main part, and, if performed, cool-down and/or additional individual drills). For matches, only the match playing time was considered (i.e., all activities before kick-off, during half-time, and after the end of the match were excluded). All training sessions, as defined above, that a player performed within the period of interest were included in the analysis. This means, for example, regardless of whether a player was fielded at all or for how long in the previous or next match, (30), the microcycle structure (31, 32) or situational and environmental conditions (19, 24, 33). Match data were only considered if a player was fielded for the whole time and, for the position-specific match reference values, data of a player and match were considered only if he played the entire match in the same position. Furthermore, only championship and cup matches, in which the match format was two halves of 45 min, separated by a 15-minute break, were included (i.e., matches with overtime and penalty shoot-outs were excluded). If data on a player in one of these included match observations were incomplete (due to technical or practical problems with the devices used to measure load), the observation was excluded (*n* = 2). The training sessions and matches took place on natural grass pitches and artificial turf pitches. The pitch surface in training was chosen depending on weather conditions, infrastructural conditions, and the next match.

All training sessions were classified according to the number of days before or after a match day [i.e., match day (md) minus or plus], with the assignment to a match being chosen according to the focus of a training session. For example, md-2 means a training session focused on the upcoming match and took place 2 days before match day. In training sessions classified as match day plus one (md+1), the players with little playing time (situationally defined by the coaching staff) in the preceding match trained on the pitch, while the players with more playing time followed an individual regeneration program off the pitch. The “general” label was assigned if a training session was not focused on a match (e.g., first part of the international break or pre-season). If multiple sessions were scheduled on one day, the external load was summarized for each player (later referred to as session day).

Five different playing positions were categorized (central defender, full back, central midfielder, wide midfielder, forward) (7, 22, 24). The positions in matches were defined by LA; if a player's position was judged to be unclear, the position was defined as the one he was required to take according to members of the coaching staff (personal communication). It is worth noting that when playing in a 4-4-2 diamond tactical formation, we deemed it appropriate to define all four midfielders as central midfielders given the coaching staff's intended positional interpretation of the players (personal communication). A player's position for a microcycle was defined as the position he played at the start of his next match or, in the case that there was no next match, the last one he played.

### External load

A variety of external load measures were monitored for each player using global navigation satellite system (GNSS) technology (Apex Pro, STATSports, Newry, Ireland) with 10 Hz sampling. The validity and reliability of the STATSports Apex 10 Hz system were previously reported elsewhere (34–36). Apex 10 Hz is a multi-GNSS augmented unit, capable of acquiring and tracking multiple satellite systems (e.g., GPS, GLONASS, Galileo, BeiDou) concurrently to provide the best possible position information. The Apex GNSS model reports information about the number of satellites connected (*M* = 15.1, *SD* = 1.8, range 10 to 22), which was lower than reported in previous literature (6, 34, 35). The Apex units present the following characteristics: 30 mm (wide) × 80 mm (high) dimensions, 48 g weight, 100 Hz gyroscope, 100 Hz tri-axial accelerometer, and 10 Hz magnetometer. For each player, an Apex unit was placed, according to the manufacturer's instructions, on the upper back between the right and left scapula through a vest. After data collection on the pitch, the Sonra software (Sonra 4.0, STATSports, Newry, Ireland) was used to download all data recorded by the GNSS and precisely define the session of each player (i.e., in training: from the beginning of the official warm-up to the end of the last drill; in matches: the respective playing time). The data was then exported as a csv file for further analysis. To avoid inter-unit errors, players used the same GNSS unit for each session, inserted in a manufacturer-provided vest (10).

The 11 external load measures listed in Table 1 were selected for analysis. The distance-related measures, accelerations, and decelerations, all with their respective thresholds, were selected because they have been used most frequently in practice and in studies analyzing external load (especially in soccer), and the literature suggests that they be considered (12, 14, 37, 38). The latter also applies to total loading and total time. Despite certain concerns, which should be taken into account with high metabolic load (HML) distance [m] and dynamic stress load [e.g. (39)], these two measures were also assessed as they might contribute to a more complete picture to some extent and are also regularly used (12, 18, 40, 41). The percentage thresholds applied for the relative speed thresholds (i.e., 55 and 70) are explained by the fact that they correspond to the recommended fixed thresholds (12) for a maximum speed of 36 km/h. In the present analysis, the individual maximum speed was defined as the respective highest speed measured by GNSS (42), provided it followed a proper acceleration phase, the absence of which reveals clear measurement errors. In the case of a new maximum speed being measured, the new value replaced the previous one.

**Table 1:** Definitions and units of the external load measures included in the analysis.

External load measure	Unit	Definition
Accelerations	[n]	Acceleration efforts performed between 4 and 10 m/s^2^ with a minimum duration of 0.5 s
Decelerations	[n]	Deceleration efforts performed between 4 and 10 m/s^2^ with a minimum duration of 0.5 s
Dynamic stress load	[a.u.]	The total of the weighted impacts, which is based on accelerometer values of magnitude above 2 g. This measure weights the impacts using a convex-shaped function. The aggregated weighted impacts are scaled to provide more workable values.
High metabolic load distance	[m]	The distance [m] covered by a player performing any activity with a metabolic power (energy consumption per kilogram per second) of ≥25.5 W/kg for at least 1 s.
High-speed distance		
Absolute	[m]	Distance ≥19.8 km/h (5.5 m/s).
Relative	[m]	Distance ≥55% of individual maximal speed.
Sprint distance		
Absolute	[m]	Distance ≥25.2 km/h (7 m/s).
Relative	[m]	Distance ≥70% of individual maximal speed.
Total distance	[m]	Total distance covered.
Total loading	[a.u.]	The total force on the player over the entire session based on accelerometer data alone and without any weightings. It uses the magnitude of the accelerometer values taken in three directions, sampled with 100 Hz. The total is scaled by 1,000 to provide more workable values.
Total time	[min]	Total session time.

a.u., arbitrary units*.*

### Statistical analyses

All data were analyzed with the open-source software RStudio (R version 4.2.2 (2022-10-31 ucrt), R Core Team (43), Wien, Austria). Descriptive statistics were used to describe and characterize the sample; thereby, the mean (SD) and range were reported. For all analysis referring to days in a microcycle, only days with a focus on a match were presented (i.e., no session days with the “general” label). The mean and standard error were calculated for the load analysis in absolute numbers for each load measure per player and day in a microcycle. Coefficients of variations (standard deviation divided by mean; expressed as a percentage) were calculated per player and day in a microcycle to assess the load variation that players experienced within a day in a microcycle.

For the ratio analysis, first, a match mean was calculated (matches included as described above), by player in the player-specific analysis and by position in the position-specific analysis. These match reference values were then used to calculate a training match ratio for each player's session day as follows: session day load multiplied by 100 and divided by match reference load. From this, the ratio mean and standard error of each load measure were calculated per day in a microcycle for each player in the player-specific analysis and for each player-position combination in the position-specific analysis. To assess the differences between these two ratios, first, the player-position-specific ratio was subtracted from the respective player-specific ratio for each player's session day, always applying the actual player position. From these differences, a mean value was then calculated by player and day in a microcycle.

## Results

Taking the data from each participant's last session in the data set, the average height of the 20 professional soccer players was =1.819 m (*SD* = 0.051 m, range = 1.73 to 1.95 m) and the average body weight was = 76.88 kg (*SD* = 6.45 kg, range = 67.0 to 89.4 kg). The mean age of the participants was = 26.29 years (*SD* = 4.43 years, range = 19.4 to 37.2 years) and an average individual maximal speed of = 33.930 km/h (*SD* = 1.440 km/h, range = 31.60 to 37.40 km/h) was recorded.

Table 2 shows the distribution of the session days per player across the days of a microcycle for the two respective analysis approaches, player-specific and position-specific. Furthermore, the total number of session days across the days of a microcycle as well as the number of matches per position used to calculate position-specific match reference values are presented. The number of individual match observations per playing position being determined by tactical formations chosen by the coaches and the substitutions they made; our data indicate that substitutions involved offensive positions more often than defensive positions. Furthermore, tactical formations with wide midfielders were rarely chosen and/or wide midfielders were rarely fielded for an entire match.

**Table 2:** Distribution of the session days per player across the days of a microcycle for the two respective analysis approaches.

	md-5 (*n* = 92)	md-4 (*n* = 329)	md-3 (*n* = 398)	md-2 (*n* = 433)	md-1 (*n* = 674)	md	md+1 (*n* = 150)
**Player specific**						(*n* = 280)	
Mean (SD)	4.6 (2.6)	16.4 (6.4)	19.9 (8.0)	21.7 (7.7)	33.7 (11.4)	14.0 (12.5)	7.9 (7.4)
Range	1 to 8	5 to 27	5 to 33	8 to 34	12 to 50	3 to 48	1 to 27
**Position specific**						def cent: 88 def wide: 76 mid cent: 78 mid wide: 4 forward: 14	
Mean (SD)	3.5 (2.5)	11.0 (7.9)	12.4 (10.0)	14.0 (10.3)	21.1 (15.8)		6.5 (6.6)
Range	1 to 8	1 to 27	1 to 33	1 to 34	2 to 50		1 to 27

Data presented as mean (SD), range, and total, except for position-specific match day (md) where total values are presented for each position. Def cent, central defender; def wide, full back; mid cent, central midfielder; mid wide, wide midfielder.

**Figure 1:**
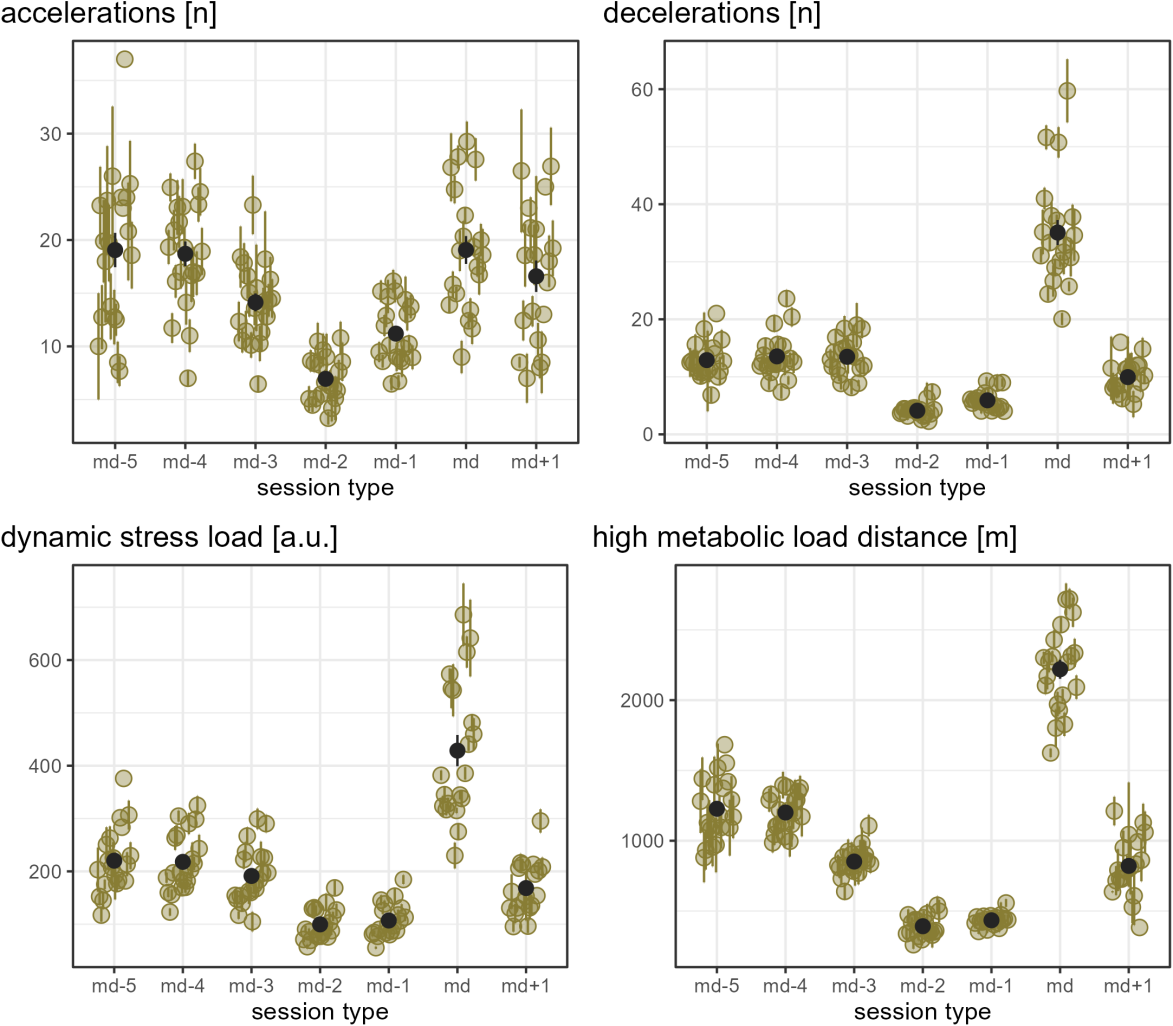
The old-moss-greenish dots indicate the mean of the respective load measure in absolute numbers by player and day in a microcycle, and the corresponding old-moss-greenish error bars range from mean minus standard error to mean plus standard error. The dark grey dots show the mean of all players' means per day in a microcycle, and the dark grey error bars range from mean minus standard error to mean plus standard error.

**Figure 2:**
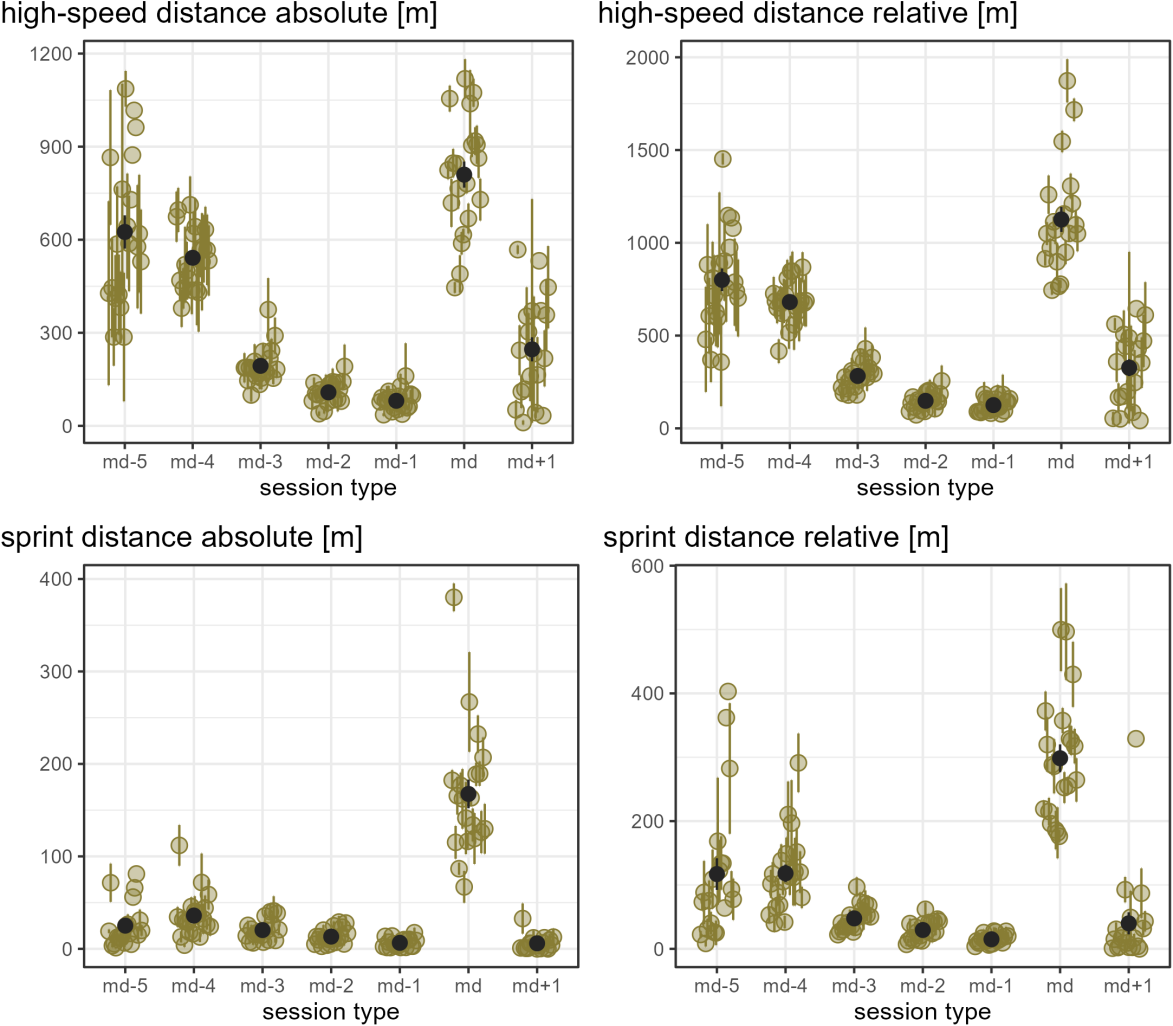
The old-moss-greenish dots indicate the mean of the respective load measure in absolute numbers by player and day in a microcycle, and the corresponding old-moss-greenish error bars range from mean minus standard error to mean plus standard error. The dark grey dots show the mean of all players' means per day in a microcycle, and the dark grey error bars range from mean minus standard error to mean plus standard error.

**Figure 3:**
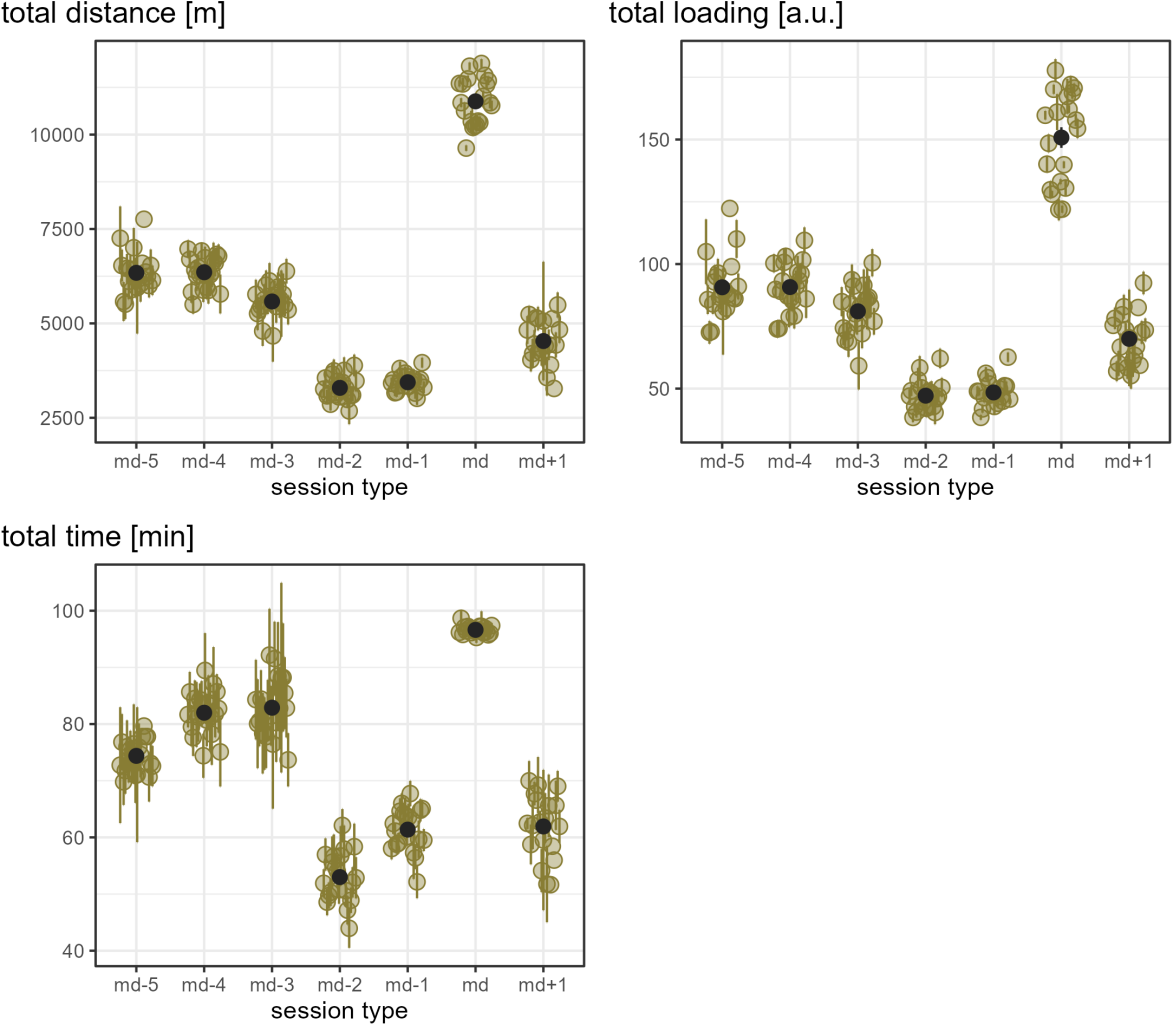
The old-moss-greenish dots indicate the mean of the respective load measure in absolute numbers by player and day in a microcycle, and the corresponding old-moss-greenish error bars range from mean minus standard error to mean plus standard error. The dark grey dots show the mean of all players' means per day in a microcycle, and the dark grey error bars range from mean minus standard error to mean plus standard error.

Figures 1–3 show the load that players experienced per day of a microcycle for each external load measure, assessed in absolute numbers. As an overall pattern of the microcycles, the load was observed to be reduced on md-2 and md-1 compared to the other days and highest on match day for all load measures except accelerations.

**Figure 4:**
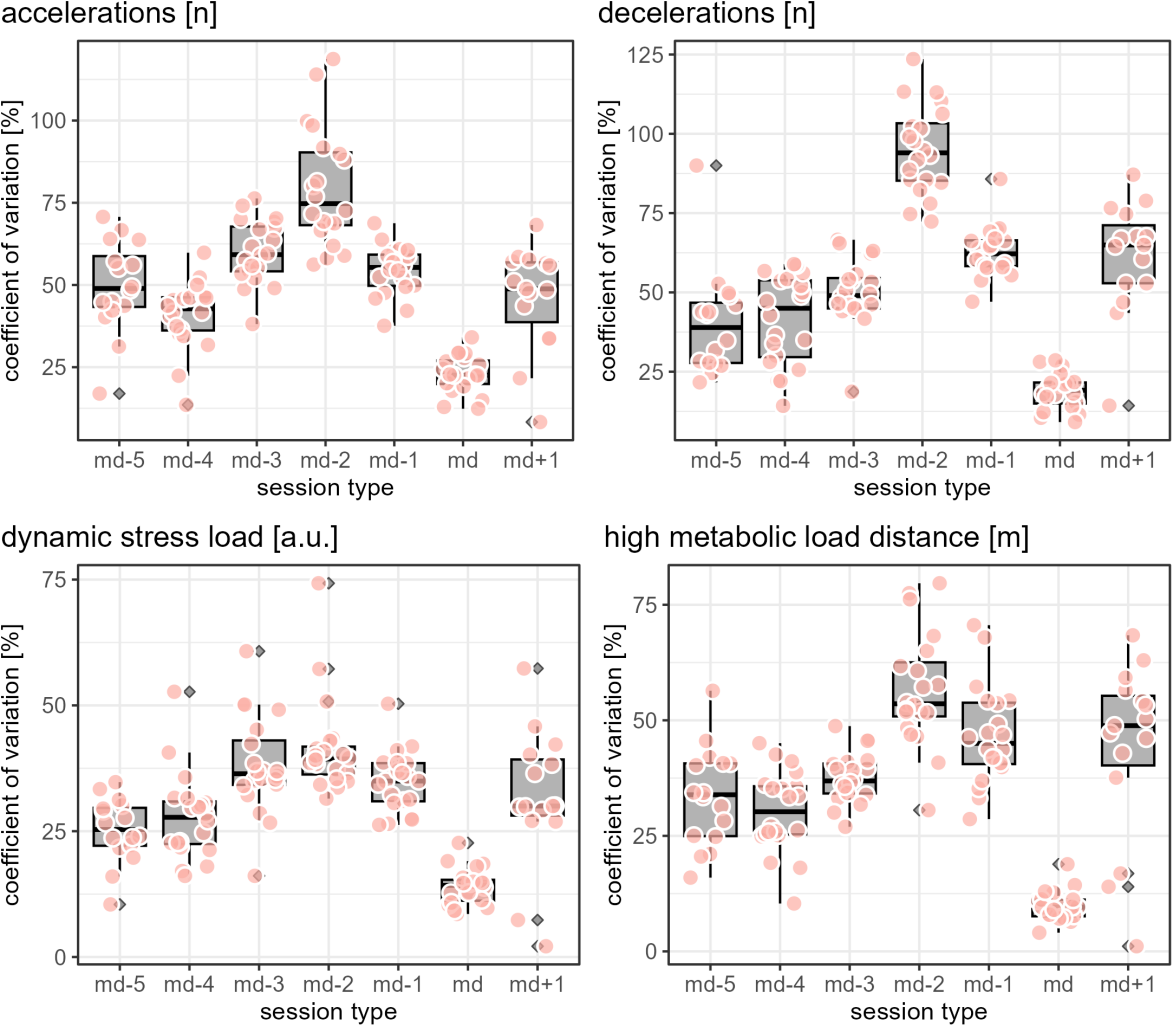
The apricot-blushed-like filled data points show the coefficient of variation by player and day in a microcycle for the respective load measure. The boxplots display the distribution of the players' coefficients of variations per day in a microcycle, with the grey filled diamonds indicating outliers (values further than 1.5 * inter-quartile range from the hinge).

**Figure 5:**
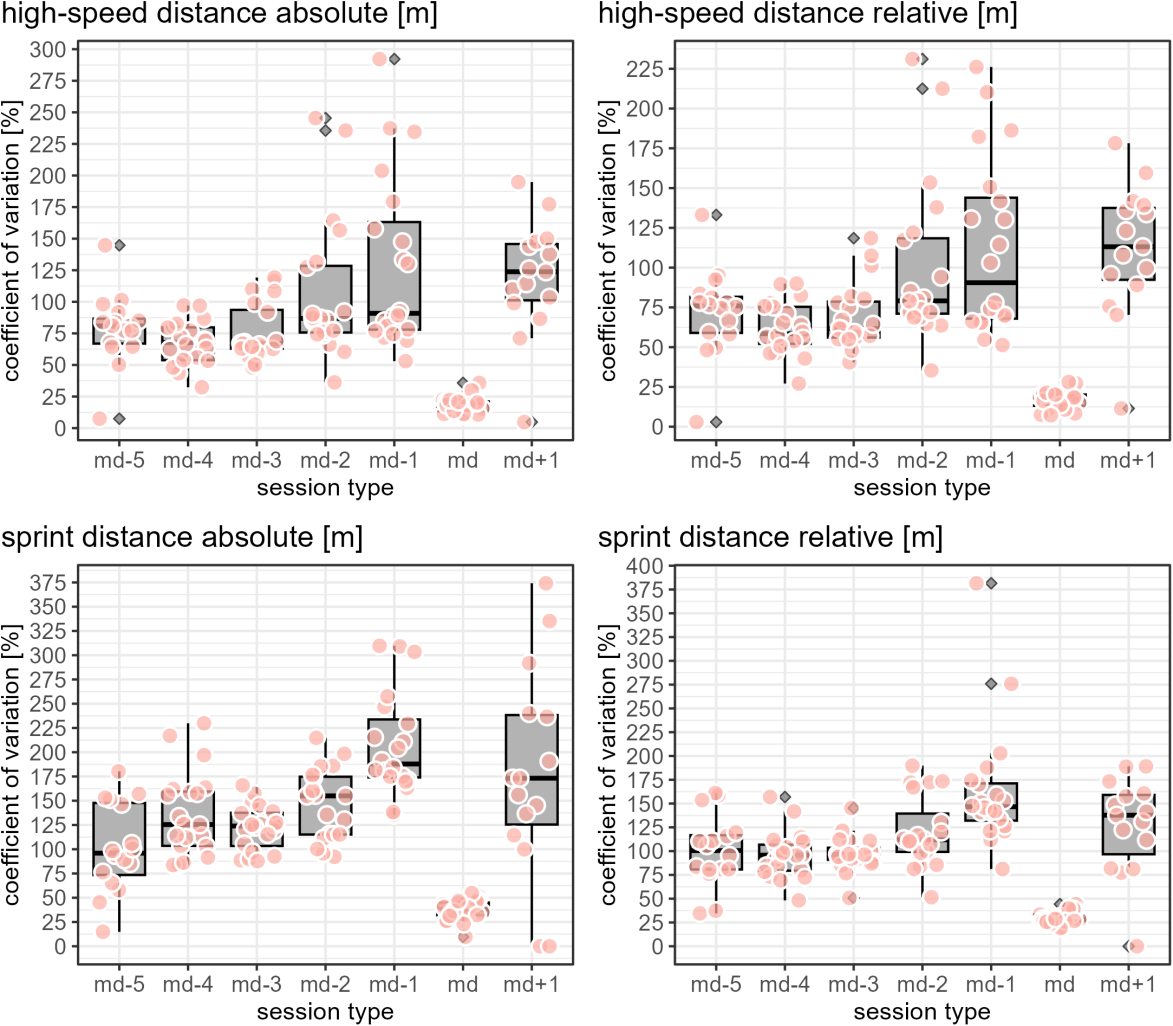
The apricot-blushed-like filled data points show the coefficient of variation by player and day in a microcycle for the respective load measure. The boxplots display the distribution of the players' coefficients of variations per day in a microcycle, with the grey filled diamonds indicating outliers (values further than 1.5 * inter-quartile range from the hinge).

**Figure 6:**
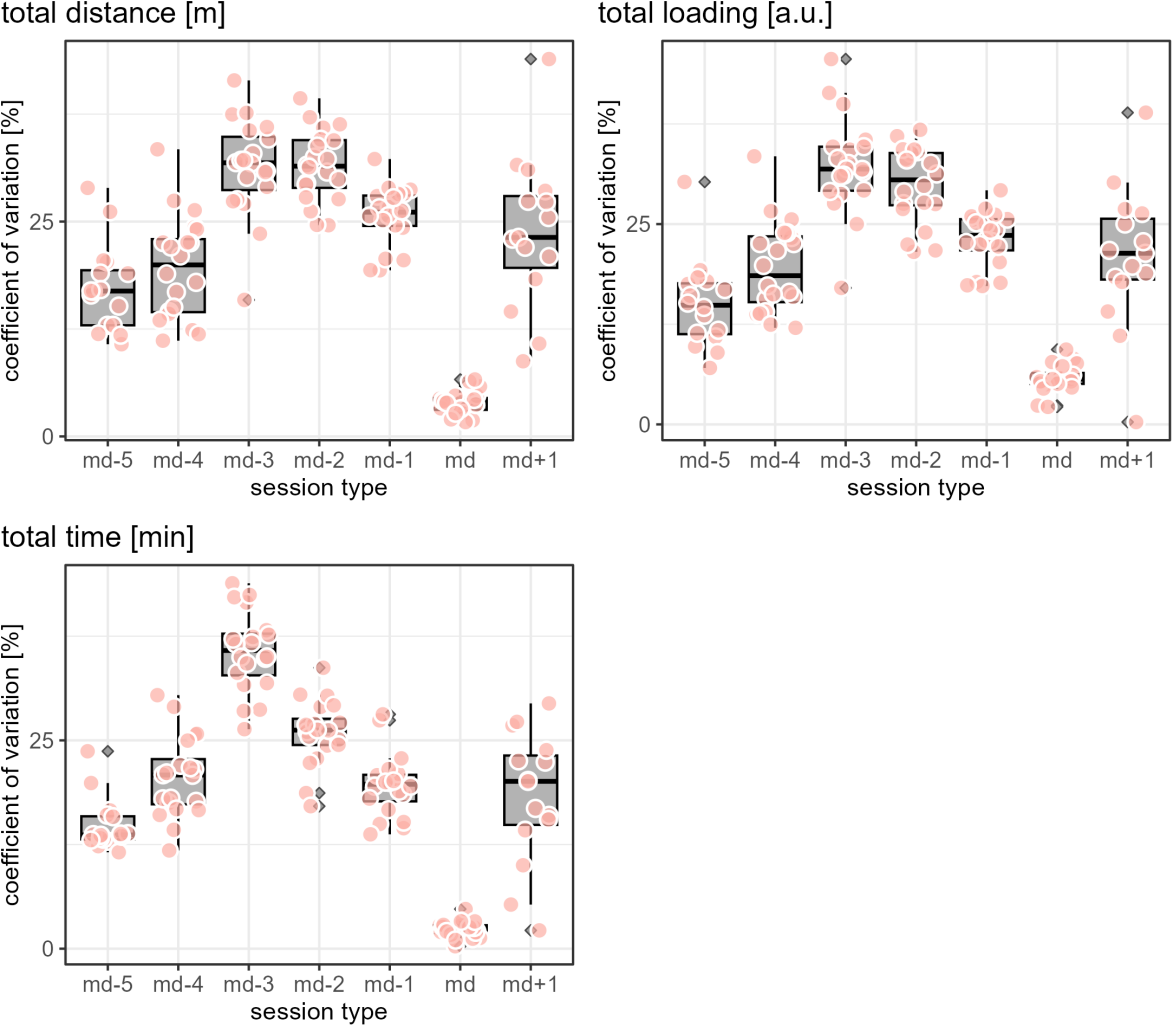
The apricot-blushed-like filled data points show the coefficient of variation by player and day in a microcycle for the respective load measure. The boxplots display the distribution of the players' coefficients of variations per day in a microcycle, with the grey filled diamonds indicating outliers (values further than 1.5 * inter-quartile range from the hinge).

While Figures 1–3 provide a rough indication of the load variation by player and day in a microcycle, Figures 4–6 do so more specifically by presenting the coefficient of variation (CV) for each load measure assessed by player and day in a microcycle. Independent of the day in a microcycle, the measures with the lowest CV were total distance, total loading, and total time. The CV increased over the measures dynamic stress load, HML distance, accelerations and decelerations followed by high-speed and sprint distance, being highest for the latter.

**Figure 7:**
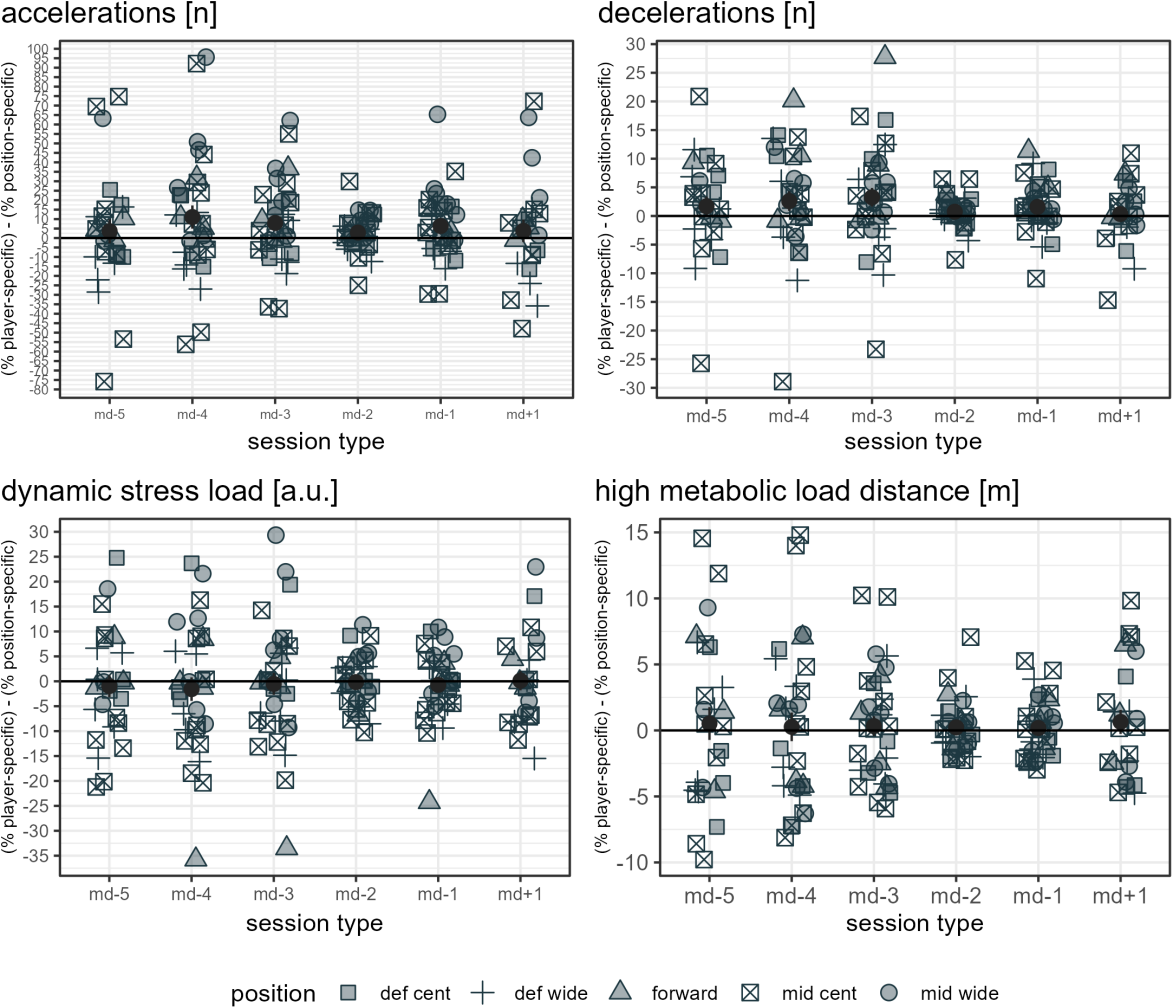
The data points listed in the figure legend show the mean observed difference between the respective player-specific ratio and the respective player-position ratio by player and day in a microcycle for the respective load measure, specifying a player's position. The dark grey dots indicate the mean of all players' mean differences per day in a microcycle, and the dark grey error bars range from mean minus standard error to mean plus standard error. Def cent, central defender; def wide, full back; mid cent, central midfielder; and mid wide, wide midfielder.

**Figure 8:**
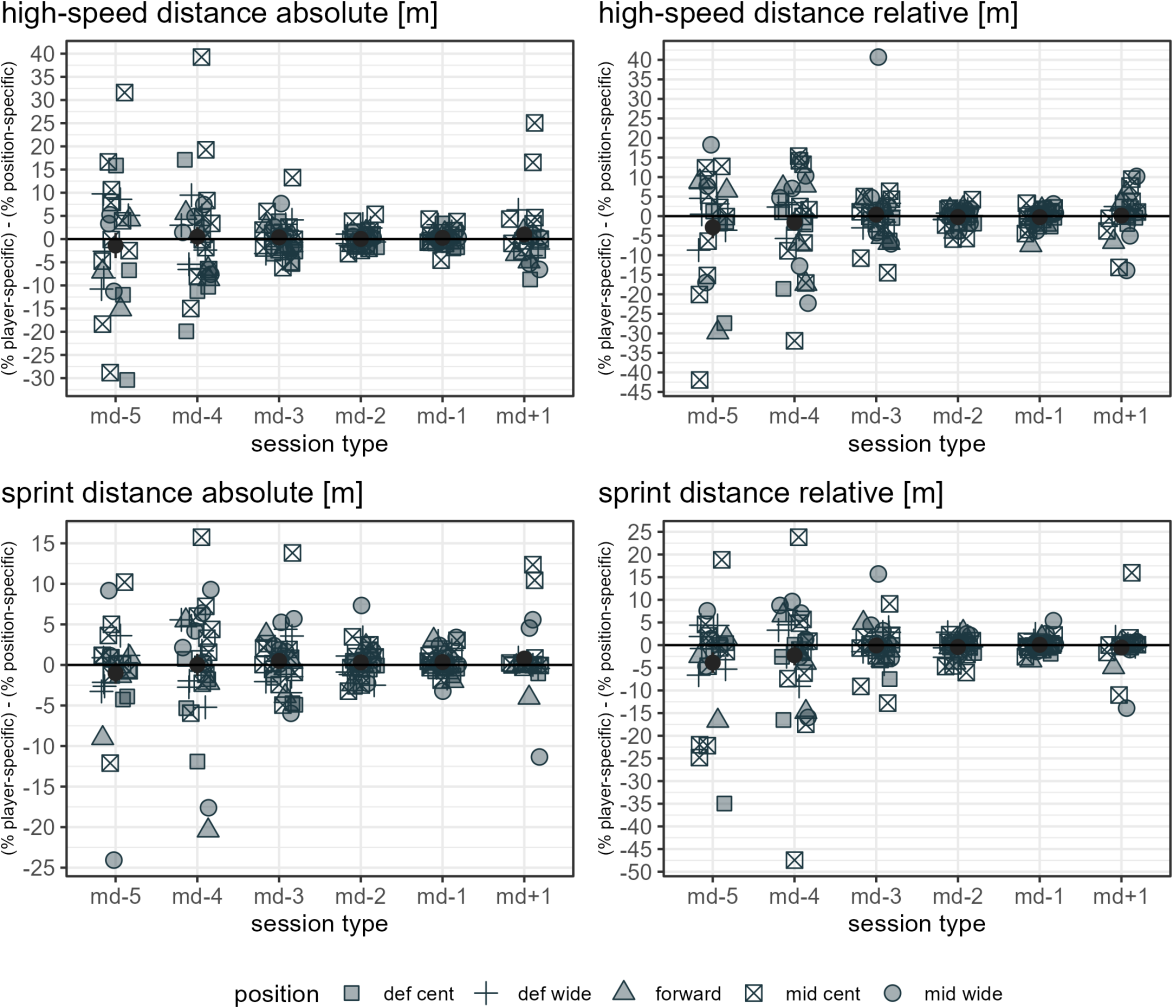
The data points listed in the figure legend show the mean observed difference between the respective player-specific ratio and the respective player-position ratio by player and day in a microcycle for the respective load measure, specifying a player's position. The dark grey dots indicate the mean of all players' mean differences per day in a microcycle, and the dark grey error bars range from mean minus standard error to mean plus standard error. Def cent, central defender; def wide, full back; mid cent, central midfielder; and mid wide, wide midfielder.

**Figure 9:**
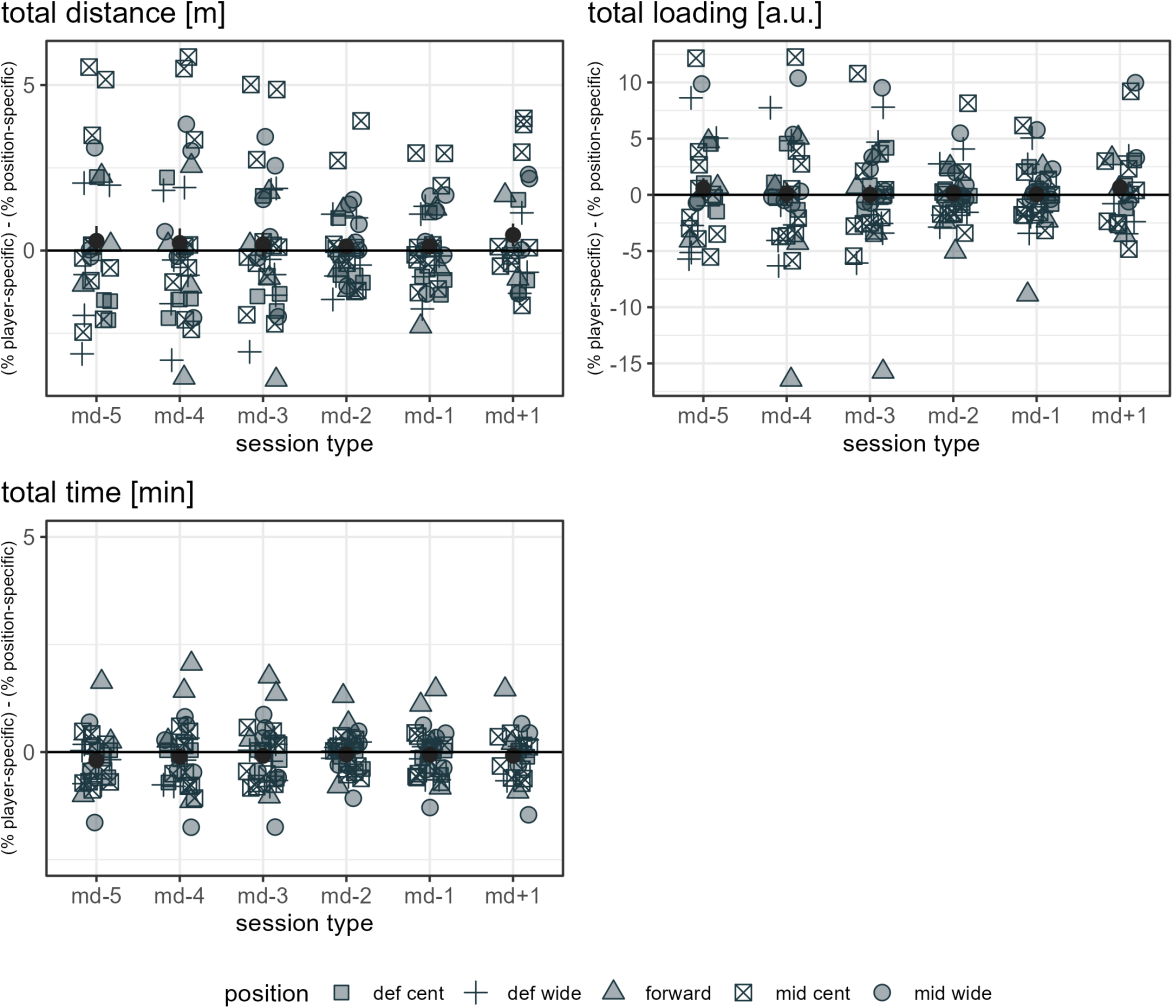
The data points listed in the figure legend show the mean observed difference between the respective player-specific ratio and the respective player-position ratio by player and day in a microcycle for the respective load measure, specifying a player's position. The dark grey dots indicate the mean of all players' mean differences per day in a microcycle, and the dark grey error bars range from mean minus standard error to mean plus standard error. Def cent, central defender; def wide, full back; mid cent, central midfielder; and mid wide, wide midfielder.

The ratio mean and standard error of each load measure assessed per day in a microcycle are presented in Supplementary Figures S1–S3 for each player and in Supplementary Figures S4–S6 for each player-position combination. Note: since multiple positions are possible for a player, more data points are given in Supplementary Figures S4–S6 compared to Supplementary Figures S1–S3 and Figures 1–3. Furthermore, since the error bars (standard error) in Figures 1–3, Supplementary Figures S1–S3 and Supplementary Figures S4–S6 provide an indication of the uncertainty of the mean, the few cases minimally reaching into negative y-axis values should not be misinterpreted to indicate negative load, which would not be possible from a practical point of view. Figures 7–9 show the mean differences between the respective player-specific ratio and the respective player-position ratio per day in a microcycle for each load measure assessed. Differences are evident in all measures. Note: since multiple positions are possible for a player, the mean difference of all observations of a day in a microcycle does not necessarily have to be zero.

## Discussion

The aim of this study was to quantify and compare the external training and match load of professional soccer players belonging to a team competing in the highest Swiss league (Credit Suisse Super League®) per day of a microcycle. Specifically, training and match load was analyzed in absolute numbers as well as by expressing training load as a percentage of match load, with the latter being player-specific and position-specific, respectively. Furthermore, the load variation that players typically experience per day in a microcycle was assessed.

### Load distribution over a microcycle and within days of a microcycle

The given larger number of registered player session days as the match day approaches (Table 2) may be explained by several reasons. In a standard one-match-per-week microcycle, md-5 was often a day off. Furthermore, there were a few weeks with more than one match per week scheduled, reducing the number of training days in between. The number of observations on md+1 is smaller since there were multiple microcycles without md+1 sessions and, as mentioned above, if there were sessions on the pitch on md+1, only players with little playing time in the preceding match were present.

The present data support the existing literature showing training in professional soccer being planned predominantly in microcycles (18, 19, 44). The observed pattern of training load, in general, being highest on md-5 and md-4, reduced on md-3 and md+1, and lowest on md-2 and md-1 was also similarly reported in other populations, though there is no consensus on the training day with the highest load (14, 18, 31, 32, 44, 45). In the present observation, md+1 sessions were typically intended to compensate for reduced match load (personal communication). This has also been reported in other soccer populations (18, 46). As there were multiple microcycles without md+1, the question arises, and cannot be answered satisfactorily with the analysis done here, as to whether, in such cases, coaches ensured otherwise that players with a low match load experienced their required training stimuli.

As expected, substantial differences in typical match load were evident both between individual players (player-specific) and between the assessed player positions. Furthermore, training session days revealed large inter-individual load differences. For example, in matches, player-specific total distance ranged from 9642 m to 11,884 m, and player-specific absolute sprint distance from 67 m to 380 m, and while one player never covered more than 40 m of relative sprint distance on all microcycle training days, another player typically covered up to 403 m on one day of a microcycle. Taken together, and considering that players usually have different strengths and weaknesses and, moreover, do not respond uniformly to similar stimuli, these observations underpin the importance of individualized load management even within team sports.

The finding of the present observation of the CV for load to be highly associated with the day in a microcycle and the load measure is in accordance with the results of Martin-Garcia et al. (18), who also add the playing position. In the investigation mentioned, high-speed running and sprint distance were the measures showing the most variability within a microcycle. Likewise, Guerrero-Calderon et al. (23) analyzed several running-based measures similar to those studied in this observation in matches and found that the higher the running speed, the higher the coefficients of variation. Total distance, total loading, and total time in training being the measures with the lowest CV could indicate that the coaches primarily paid attention to total distance and time when trying to control training load and had a certain fixed idea about them, i.e., target values which either did not vary or varied only slightly. Interestingly, the other eight measures assessed (accelerations, decelerations, dynamic stress load, HML distance, high speed, and sprint distances) in the present observation seemed to tend toward higher load variation on md-2 and md-1 compared to the other days in a microcycle. This suggests another assumption, which is that on these two days, the coaches adjusted the training sessions more to actual situational aspects, especially the expected challenges of the next match and the corresponding (tactical) ideas, whereas on the other days, they followed a more routine program.

### Training match load ratios

In line with existing literature (18, 20, 32), a typical full match presented a unique load for players within a microcycle. Of the measures assessed, the mean load plus respective standard deviation was not equal to or greater than the average match load for any player on any day in a microcycle for decelerations, HML distance, absolute sprint distance, and total distance. On the contrary, the number of accelerations performed on average on a training day other than md-2 were similar to or higher than their personal typical match load for several players. These comparatively large numbers of accelerations in training sessions may be attributed to exercises performed in relatively small areas (18, 20, 47), which were included regularly in the team under investigation (personal communication). Interestingly, a few players covered a high-speed distance comparable to what they or their reference position typically did in a match on md-5 and md-4, while this was rarely observed on md+1.

From a theoretical point of view, differences between player-specific and position-specific ratios refer to the degree to which player performance does not coincide with positional reference. Player-specific and position-specific ratios differed the most for accelerations, with some observed mean ratio differences being up to approximately 100%. While for total distance and total time, the mean observed ratio differences were in a range below approximately 5% when player-specific and position-specific references were applied, and for HML distance and total loading in a range up to approximately 15%, clearer differences (up to ca. 25% to 40%) were registered for decelerations, dynamic stress load, high-speed distance, and sprint distance, especially on md-5, md-4, md-3, and md+1. Note that, because of the different units, the same percentage difference for two measures does not imply an identical difference in load.

For illustration purposes, two examples, which we consider to be well representative (e.g., no extreme cases, regular players), from our data follow. There was one player whose player-specific absolute sprint distance match reference was 232 meters, and the corresponding position-specific match reference was 163 meters. Assuming there was a training session in which he covered 40 meters of absolute sprint distance, the observed training match load ratios would be 17.24% and 24.54%, respectively. Put another way, to finish the session with 24.54% of his individual typical match load, he would have had to sprint 17 meters more – which is almost half of what he did. For another player, a player-specific match reference of 11,011 meters total distance was registered. If he had covered 5,500 meters in a training session, this would be 49.95% of his individual match reference and 51.34% of the corresponding position-specific match reference (10,713 meters), i.e., depending on which of the two match references presented is applied, the training match load ratio observed is 1.39% higher or lower. If the aforementioned player had finished the session with 49.95% of the position-specific match reference, he would have covered 149 meters less.

The striking differences identified indicate that whenever ratios are built, one should be aware of the aim and select appropriate reference values depending on it. Only regarding the included match data, there are many factors that should be taken into account (e.g., minimum number of observations, competition format, ball in play time). Both ratios of measures with relatively large percentage deviations and ratios of measures with relatively small percentage differences require accurate interpretation to draw adequate conclusions for load management.

### Limitations, future direction

In general, caution must be taken when generalizing the current findings, since data from only one team were analyzed. It should also be noted that the conclusions are not based on inferential statistical results. However, and while it can be argued pro or con in purely fundamental terms, the methodology chosen was considered the most appropriate in the conception of the study design.

Since it was only possible to collect internal load data (e.g., sRPE training load, heart rate-based measures) from a reduced number of sessions, the present work focuses on external load. Nevertheless, we consider it important to emphasize here, again, that an integrated load monitoring approach should be followed. In the current analysis, position-specific match reference values are based on the players of the team under observation. While this might reflect the playing philosophy of the coach, one could also argue for the use of broader references (e.g., league), as it could be that players of the team, although fielded for the entire duration of the match, do not perform as required to be competitive. Furthermore, we did not set a threshold of a minimal number of session days per day in a microcycle required for a player (-position combination) to be included in the analysis. While doing so might have reduced susceptibility to outliers, it would have further reduced the sample and thus potentially skewed the overall impression of the team.

While there is no doubt that accelerations and decelerations should be considered in professional load management (12, 47–49), it is less clear how to quantify such loads (47, 49). As it is common in various studies and recommended in the literature, we employed threshold-based counts (12, 48, 49). However, regarding accelerations, Sonderegger et al. (50) showed that if the running speed immediately prior to an acceleration being initiated and the maximal acceleration capacity associated with it are not considered, a number of high-intensity accelerations could be missed, i.e., arbitrarily set thresholds lead to accelerations from low speeds being overestimated and accelerations from high speeds being underestimated.

## Conclusion

The present data support the existing literature that shows training in professional soccer being planned predominantly in microcycles. Therein, typical full matches present a unique load for players, and training loads prove to be higher on days with a greater temporary distance to a match or on the day directly following a match. Load variation was demonstrated to be highly associated with the day in a microcycle and the load measure. Substantial differences in typical load were evident for players in trainings as well as in matches, both between individual players and between the assessed player positions. Taken together, and considering that players usually have different strengths and weaknesses and, moreover, do not respond uniformly to similar stimuli, the present data underpin the importance of individualized load management, even within team sports. When consulting training match load ratios for that purpose, one should be aware of the aim and select appropriate reference values depending on it, as the present analysis reveals striking differences when applying player-specific or position-specific match reference values. Furthermore, all ratios require accurate interpretation to draw adequate conclusions.

## Data Availability

The datasets presented in this article are not readily available because in combination with free available data, the datasets allows identification of the participants. Requests to access the datasets should be directed to linda_ammann@gmx.ch.

## Supplementary material

Supplementary Figure S1–S3 The caramel-colored dots indicate the ratio mean of the respective load measure by player and day in a microcycle, and the caramel-colored error bars range from mean minus standard error to mean plus standard error. The dark gray dots show the mean of all players ratio means per day in a microcycle, and the dark gray error bars range from mean minus standard error to mean plus standard error.

Supplementary Figure S4–S6 The green dots indicate the ratio mean of the respective load measure by player and day in a microcycle, and the green error bars range from mean minus standard error to mean plus standard error. The dark gray dots show the mean of all players ratio means per day in a microcycle, and the dark gray error bars range from mean minus standard error to mean plus standard error.

## References

[1] Dambroz F Clemente FM Teoldo I. The effect of physical fatigue on the performance of soccer players: a systematic review. PLoS One. (2022) 17(7):e0270099. 10.1371/journal.pone.0270099 35834441 PMC9282585

[2] Forcher L Forcher L Wäsche H Jukauc D Woll A Altmann S. The influence of tactical formation on physical and technical match performance in male soccer: a systematic review. Int J Sports Sci Coach. (2022). Advance online publication. 10.1177/1747954122110136335663129

[3] Julian R Page RM Harper LD. The effect of fixture congestion on performance during professional male soccer match-play: a systematic critical review with meta-analysis. Sports Med. (2021) 51(2):255–73. 10.1007/s40279-020-01359-9 33068272 PMC7846542

[4] Sarmento H Clemente FM Afonso J Araujo D Fachada M Nobre P Match analysis in team ball sports: an umbrella review of systematic reviews and meta-analyses. Sports Med – Open. (2022) 8(1):66. 10.1186/s40798-022-00454-7 35553279 PMC9100301

[5] Impellizzeri FM Menaspà P Coutts AJ Kalkhoven J Menaspà MJ. Training load and its role in injury prevention, part I: back to the future. J Athl Train. (2020) 55(9):885–92. 10.4085/1062-6050-500-19 32991701 PMC7534945

[6] Ammann L Altmann S Ruf L Sperlich B. Seasonal analysis of match load in professional soccer players: an observational cohort study of a Swiss U18, U21 and first team. Front Physiol. (2023) 13:1023378. 10.3389/fphys.2022.1023378 36685210 PMC9846105

[7] Forcher L Forcher L Jekauc D Woll A Gross T Altmann S. Center backs work hardest when playing in a back three: the influence of tactical formation on physical and technical match performance in professional soccer. PLoS One. (2022) 17(3):e0265501. 10.1371/journal.pone.0265501 35298531 PMC8929644

[8] Harkness-Armstrong A Till K Datson N Myhill N Emmonds S. A systematic review of match-play characteristics in women’s soccer. PLoS One. (2022) 17(6):e0268334. 10.1371/journal.pone.0268334 35771861 PMC9246157

[9] Palucci Vieira LH Carling C Barbieri FA Aquino R Santiago PRP. Match running performance in young soccer players: a systematic review. Sports Med. (2019) 49(2):289–318. 10.1007/s40279-018-01048-8 30671900

[10] Bourdon PC Cardinale M Murray A Gastin P Kellmann M Varley MC Monitoring athlete training loads: consensus statement. Int J Sports Physiol Perform. (2017) 12(2):161–70. 10.1123/IJSPP.2017-0208 28463642

[11] Impellizzeri FM Marcora SM Coutts AJ. Internal and external training load: 15 years on. Int J Environ Res Public Health. (2019) 14(2):270–3. 10.1123/ijspp.2018-0935 30614348

[12] Miguel M Oliveira R Loureiro N Gracia-Rubio J Ibanez SJ. Load measures in training/ match monitoring in soccer: a systematic review. Int J Environ Res Public Health. (2021) 18(5):2721. 10.3390/ijerph18052721 33800275 PMC7967450

[13] Halson SL. Monitoring training load to understand fatigue in athletes. Sports Med. (2014) 44(2):139–47. 10.1007/s40279-014-0253-z PMC4213373 25200666

[14] Teixeira JE Forte P Ferraz R Leal M Ribeiro J Silva AJ Monitoring accumulated training and match load in football: a systematic review. Int J Environ Res Public Health. (2021) 18(8):3906. 10.3390/ijerph18083906 33917802 PMC8068156

[15] Ammann L Ruf L Beavan A Chmura P Altmann S. Advancing and critical appraisal of an integrative load monitoring approach in microcycles in soccer [Manuscript submitted for publication]. Department of Sport Science, University of Würzburg (2023).

[16] Lima-Alves A Claudino JG Boullosa D Couto CR Teixeira-Coelho F Pimenta EM. The relationship between internal and external loads as a tool to monitor physical fitness status of team sport athletes: a systematic review. Biol Sport. (2022) 39(3):629–38. 10.5114/biolsport.2022.107021 35959321 PMC9331329

[17] Akenhead R Nassis GP. Training load and player monitoring in high-level football: current practice and perceptions. Int J Sports Physiol Perform. (2016) 11(5):587–93. 10.1123/ijspp.2015-0331 26456711

[18] Martin-Garcia A Gomez Diaz A Bradley PS Morera F Casamichana D. Quantification of a professional football team’s external load using a microcycle structure. J Strength Cond Res. (2018) 32(12):3520–7. 10.1519/JSC.0000000000002816 30199452

[19] Oliva-Lozano JM Rago V Fortes V Muyor JM. Impact of match-related contextual variables on weekly training load in a professional soccer team: a full season study. Biol Sport. (2022) 39(1):125–34. 10.5114/biolsport.2021.102927 35173371 PMC8805347

[20] Stevens TGA de Ruiter CJ Twisk JWR Savelsbergh GJP Beek PJ. Quantification of in season training load relative to match load in professional Dutch eredivisie football players. Sci Med Footb. (2017) 1(2):117–25. 10.1080/24733938.2017.1282163

[21] Altmann S Forcher L Ruf L Beavan A Gross T Lussi P Match-related physical performance in professional soccer: position or player specific? PLoS One. (2021) 16(9):e0256695. 10.1371/journal.pone.0256695 34506520 PMC8432651

[22] Modric R Versic S Chmura P Konefal M Andrzejewski M Jukic I Match running performance in UEFA champions league: is there a worthwhile association with team achievement? Biology. (2022) 11(6):867. 10.3390/biology11060867 35741388 PMC9219775

[23] Guerrero-Calderon B Alfonso MJ Chena M Castillo-Rodriguez A. Comparison of training and match load between metabolic and running speed metrics of professional spanish soccer players by playing position. Biol Sport. (2022) 39(4):933–41. 10.5114/biolsport.2022.110884 36247950 PMC9536366

[24] Chmura P Liu H Andrzejewski M Chmura J Kowalczuk E Rokita A Is there meaningful influence from situational and environmental factors on the physical and technical activity of elite football players? Evidence from the data of 5 consecutive seasons of the German bundesliga. PLoS One. (2021) 16(3):e0247771. 10.1371/journal.pone.0247771 33690609 PMC7943014

[25] Draper G Wright MD Ishida A Chesterton P Portas MD Atkinson G. Do environmental temperatures and altitudes affect physical outputs of elite football athletes in match conditions? A systematic review of the “real world” studies. Sci Med Footb. (2022) 7(1):81–92. 10.1080/24733938.2022.2033823 35068376

[26] Hulton AT Malone JJ Clarke ND MacLaren DPM. Energy requirements and nutritional strategies for male soccer players: a review and suggestions for practice. Nutrients. (2022) 14(3):657. 10.3390/nu14030657 35277016 PMC8838370

[27] Link D de Lorenzo MF. Seasonal pacing – match importance affects activity in professional soccer. PLoS One. (2016) 11(6):e0157127. 10.1371/journal.pone.0157127 27281051 PMC4900650

[28] White A Hills SP Cooke CB Batten T Kilduff LP Cook CJ Match-play and performance test responses of soccer goalkeepers: a review of current literature. Sports Med. (2018) 48(11):2497–516. 10.1007/s40279-018-0977-2 30144021

[29] Winter EM Maughan RJ. Requirements for ethics approvals. J Sports Sci. (2009) 27(10):985. 10.1080/02640410903178344 19847681

[30] Gualtieri A Rampinini E Sassi R Beato M. Workload monitoring in top-level soccer players during congested fixture periods. Int J Sports Med. (2020) 41(10):677–81. 10.1055/a-1171-1865 32455455

[31] Anderson L Orme P Di Michele R Close GL Morgans R Drust B Quantification of training load during one-, two- and three-game week schedules in professional soccer players from the English premier league: implications for carbohydrate periodisation. J Sports Sci. (2016) 34(13):1250–9. 10.1080/02640414.2015.1106574 26536538

[32] Oliva-Lozano JM Gomez-Carmona CD Fortes V Pino-Ortega J. Effect of training day, match, and length of the microcycle on workload periodization in professional soccer players: a full-season study. Biol Sport. (2022) 39(2):397–406. 10.5114/biolsport.2022.106148 35309541 PMC8919886

[33] Oliveira R Brito JP Loureiro N Padinha V Nobari H Mendes B. Will next match location influence external and internal training load of a top-class elite professional European soccer team? Int J Environ Res Public Health. (2021) 18(10):5229. 10.3390/ijerph18105229 34069032 PMC8156245

[34] Beato M Coratella G Stiff A Iacono AD. The validity and between-unit variability of GNSS units (STATSports apex 10 hz and 18 hz) for measuring distance and peak speed in team sports. Front Physiol. (2018) 9:1288. 10.3389/fphys.2018.01288 30298015 PMC6161633

[35] Beato M de Keijzer KL. The inter-unit and inter-model reliability of GNSS STATSports apex and viper units in measuring peak speed over 5, 10, 15, 20 and 30 meters. Biol Sport. (2019) 36(4):317–21. 10.5114/biolsport.2019.88754 31938002 PMC6945047

[36] Crang ZL Duthie G Cole MH Weakley J Hewitt A Johnston RD. The inter-device reliability of global navigation satellite systems during team sport movement across multiple days. J Sci Med Sport. (2022) 25(4):340–4. 10.1016/j.jsams.2021.11.044 34893434

[37] Rago V Brito J Figueiredo P Krustrup P Rebelo A. Relationship between external load and perceptual responses to training in professional football: effects of quantification method. Sports. (2019) 7(3):68. 10.3390/sports7030068 30884900 PMC6473819

[38] Rago V Brito J Figueiredo P Krustrup P Rebelo A. Application of individualized speed zones to quantify external training load in professional soccer. J Hum Kinet. (2020) 72:279–89. 10.2478/hukin-2019-0113 32269668 PMC7126260

[39] Brochhagen J Hoppe MW. Metabolic power in team and racquet sports: a systematic review with best-evidence synthesis. Sports Med Open. (2022) 8(1):133. 10.1186/s40798-022-00525-9 36282365 PMC9596658

[40] García-Calvo T Ponce-Bordón JC Pons E López Del Campo R Resta R Raya-González J. High metabolic load distance in professional soccer according to competitive level and playing positions. PeerJ. (2022) 10:e13318. Available at: https://peerj.com/articles/13318/ 10.7717/peerj.13318 36157060 PMC9504445

[41] Reynolds J Connor M Jamil M Beato M. Quantifying and comparing the match demands of U18, U23, and 1ST team English professional soccer players. Front Physiol. (2021) 12:706451. 10.3389/fphys.2021.706451 34276425 PMC8283180

[42] Massard T Eggers T Lovell R. Peak speed determination in football: is sprint testing necessary? Sci Med Footb. (2017) 2(2):123–6. 10.1080/02640414.2015.1112023

[43] R Core Team. R: A language and environment for statistical computing. Vienna, Austria: R Foundation for Statistical Computing (2022). Available at: https://www.R-project.org/

[44] Ramos-Cano J Martín-García A Rico-González M. Training intensity management during microcycles, mesocycles, and macrocycles in soccer: a systematic review. Proceedings of the Institution of Mechanical Engineers, Part P: Journal of Sports Engineering and Technology. (2022) 0(0). 10.1177/17543371221101227

[45] Oliveira R Brito JP Martins A Mendes B Marinho DA Ferraz R In-season internal and external training load quantification of an elite European soccer team. PLoS One. (2019) 14(4):e0209393. 10.1371/journal.pone.0209393 31009464 PMC6476476

[46] Casamichana D Martín-García A Díaz AG Bradley PS Castellano J. Accumulative weekly load in a professional football team: with special reference to match playing time and game position. Biol Sport. (2022) 39(1):115–24. 10.5114/biolsport.2021.102924 35173370 PMC8805368

[47] Silva H Nakamura FY Beato M Marcelino R. Acceleration and deceleration demands during training sessions in football: a systematic review. Sci Med Footb. (2022):1–16. Advance online publication. 10.1080/24733938.2022.2090600 35700979

[48] Delves RIM Aughey RJ Ball K Duthie GM. The quantification of acceleration events in elite team sport: a systematic review. Sports Med Open. (2021) 7(1):45. 10.1186/s40798-021-00332-8 34191142 PMC8245618

[49] Harper DJ Carling C Kiely J. High-intensity acceleration and deceleration demands in elite team sports competitive match play: a systematic review and meta-analysis of observational studies. Sports Med. (2019) 49(12):1923–47. 10.1007/s40279-019-01170-1 31506901 PMC6851047

[50] Sonderegger K Tschopp M Taube W. The challenge of evaluating the intensity of short actions in soccer: a new methodological approach using percentage acceleration. PLoS One. (2019) 11(11):e0166534. 10.1371/journal.pone.0166534 PMC5112910 27846308

