# Supplementary figures and images for: Training and match load ratios in professional soccer–should we use player- or position-specific match reference values?
Source: Front Sports Act Living. 2023 May 16;5:1151828. doi: 10.3389/fspor.2023.1151828 (PMC10227614; doi:10.3389/fspor.2023.1151828)

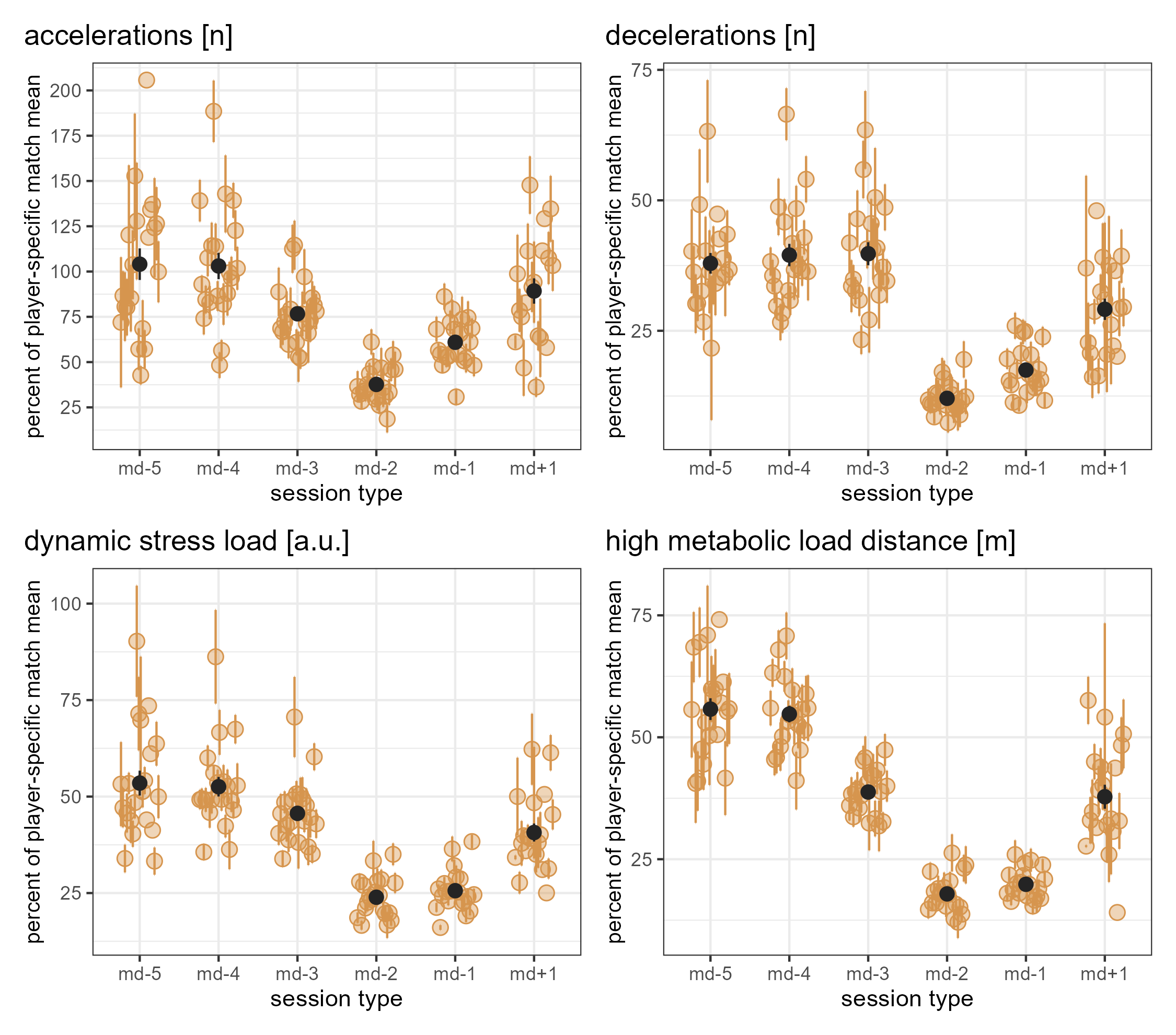

Supplement: Supplementary Figure S1–S3 — The caramel-colored dots indicate the ratio mean of the respective load measure by player and day in a microcycle, and the caramel-colored error bars range from mean minus standard error to mean plus standard error. The dark gray dots show the mean of all players ratio means per day in a microcycle, and the dark gray error bars range from mean minus standard error to mean plus standard error. [file Image1.tiff]

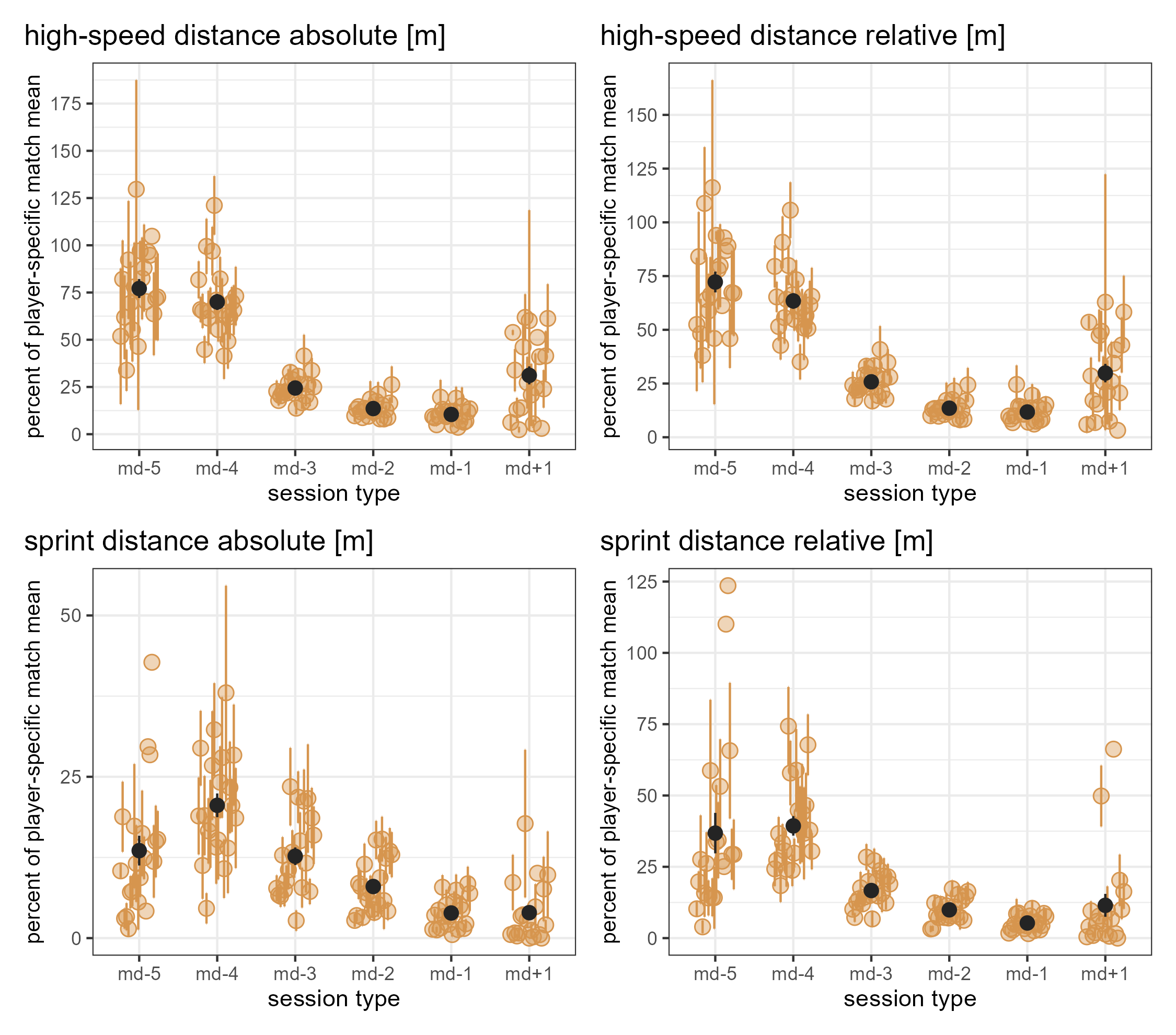

Supplement: Supplementary file 2 [file Image2.tiff]

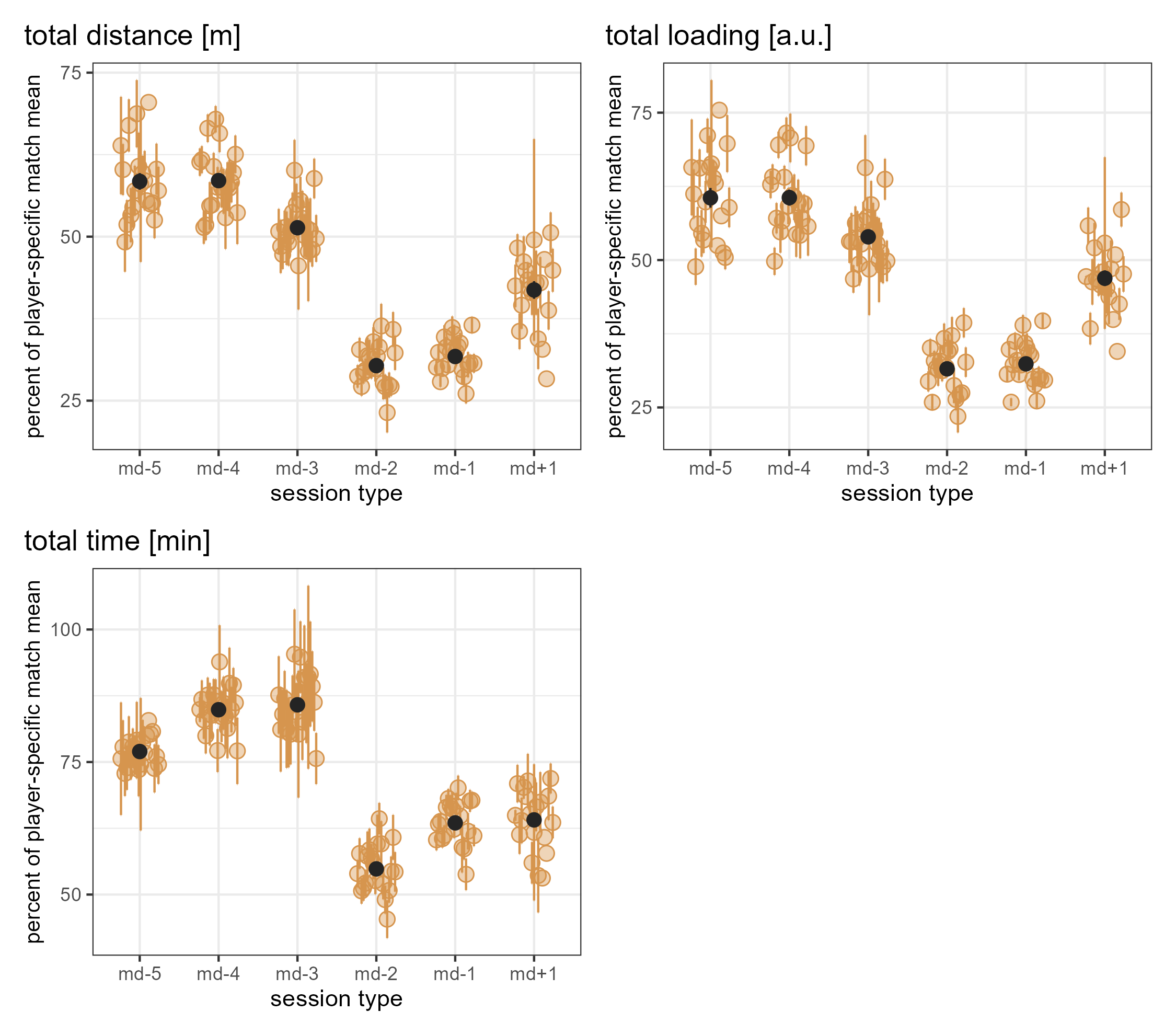

Supplement: Supplementary file 3 [file Image3.tiff]

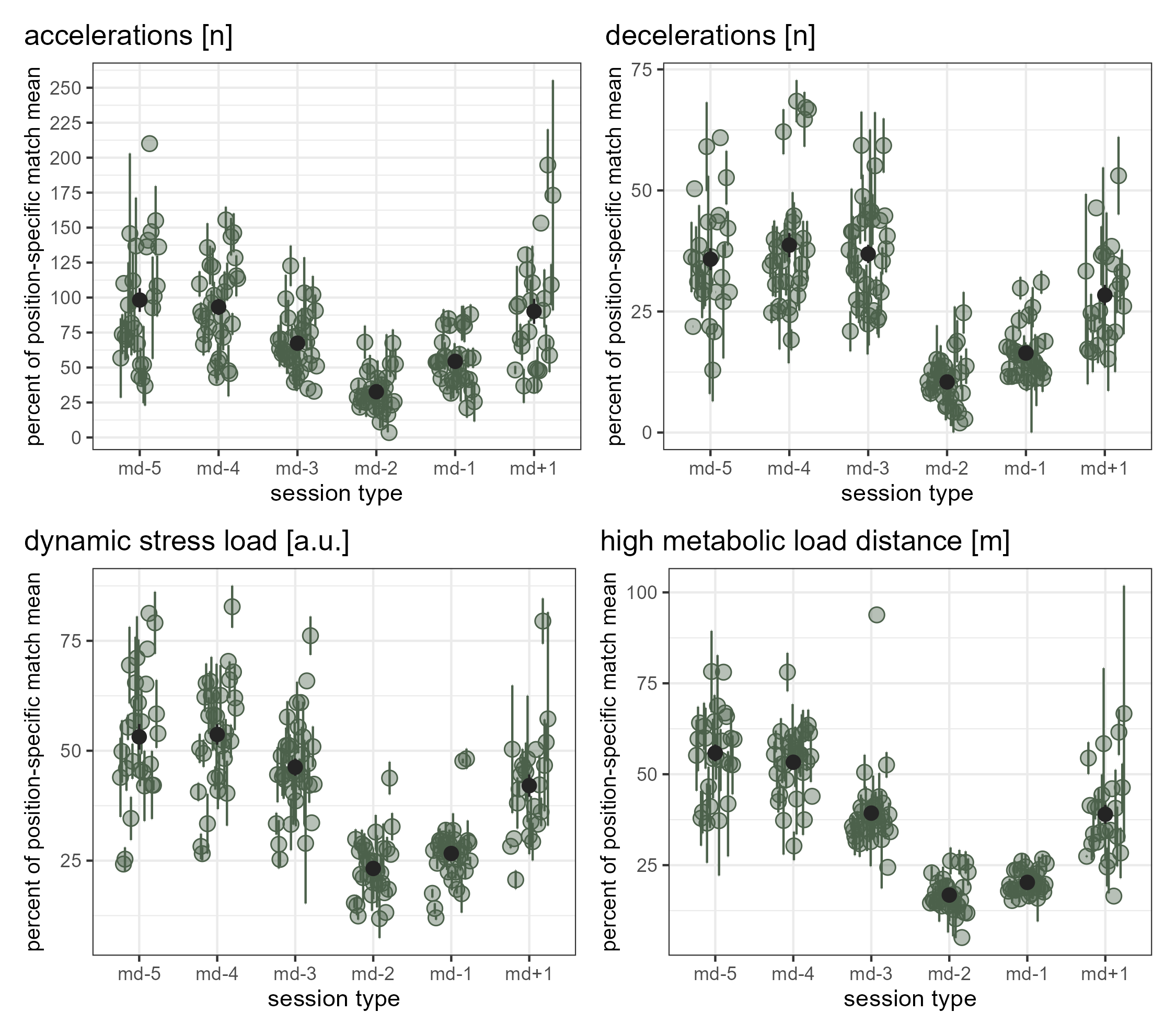

Supplement: Supplementary Figure S4–S6 — The green dots indicate the ratio mean of the respective load measure by player and day in a microcycle, and the green error bars range from mean minus standard error to mean plus standard error. The dark gray dots show the mean of all players ratio means per day in a microcycle, and the dark gray error bars range from mean minus standard error to mean plus standard error. [file Image4.tiff]

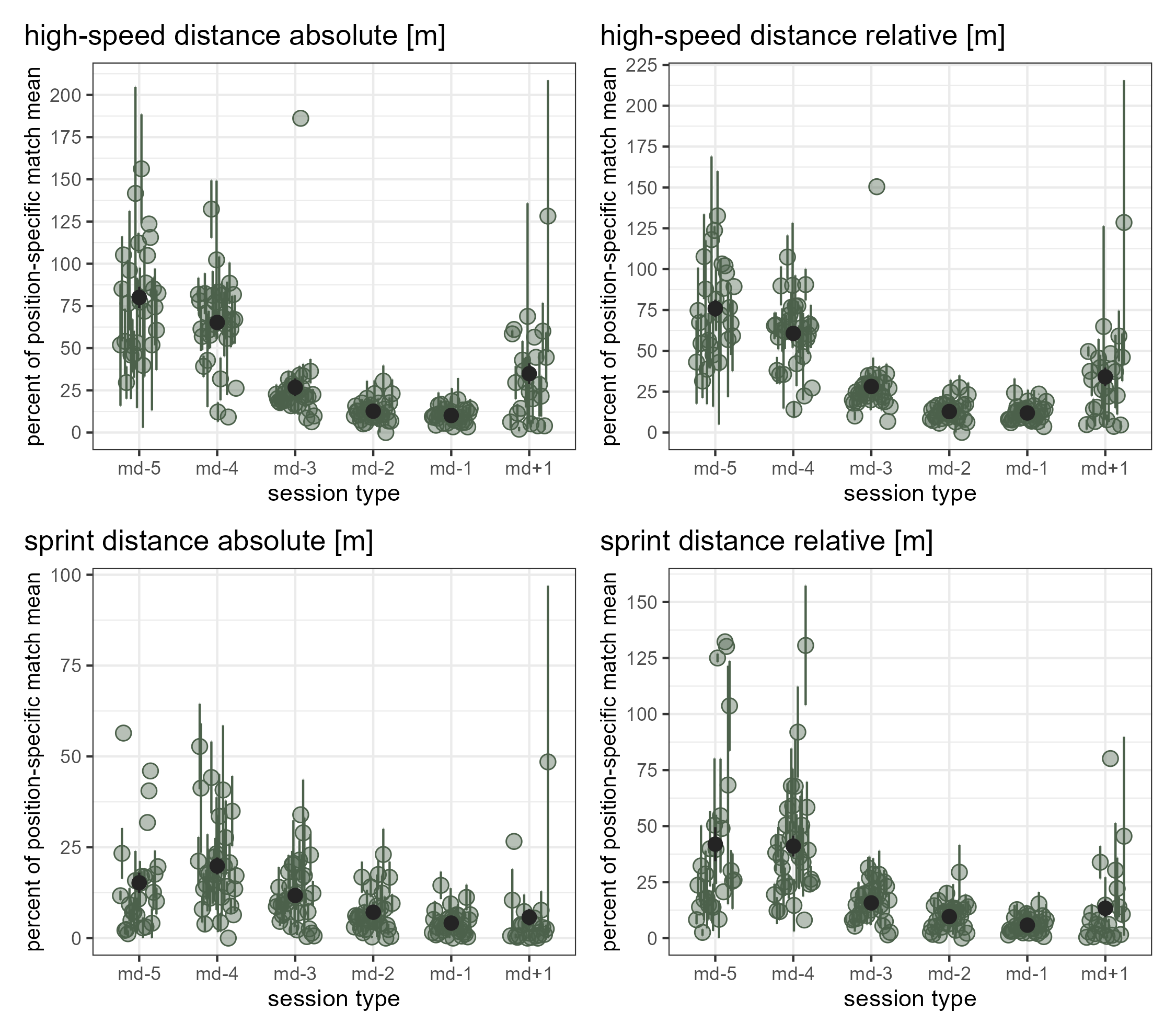

Supplement: Supplementary file 5 [file Image5.tiff]

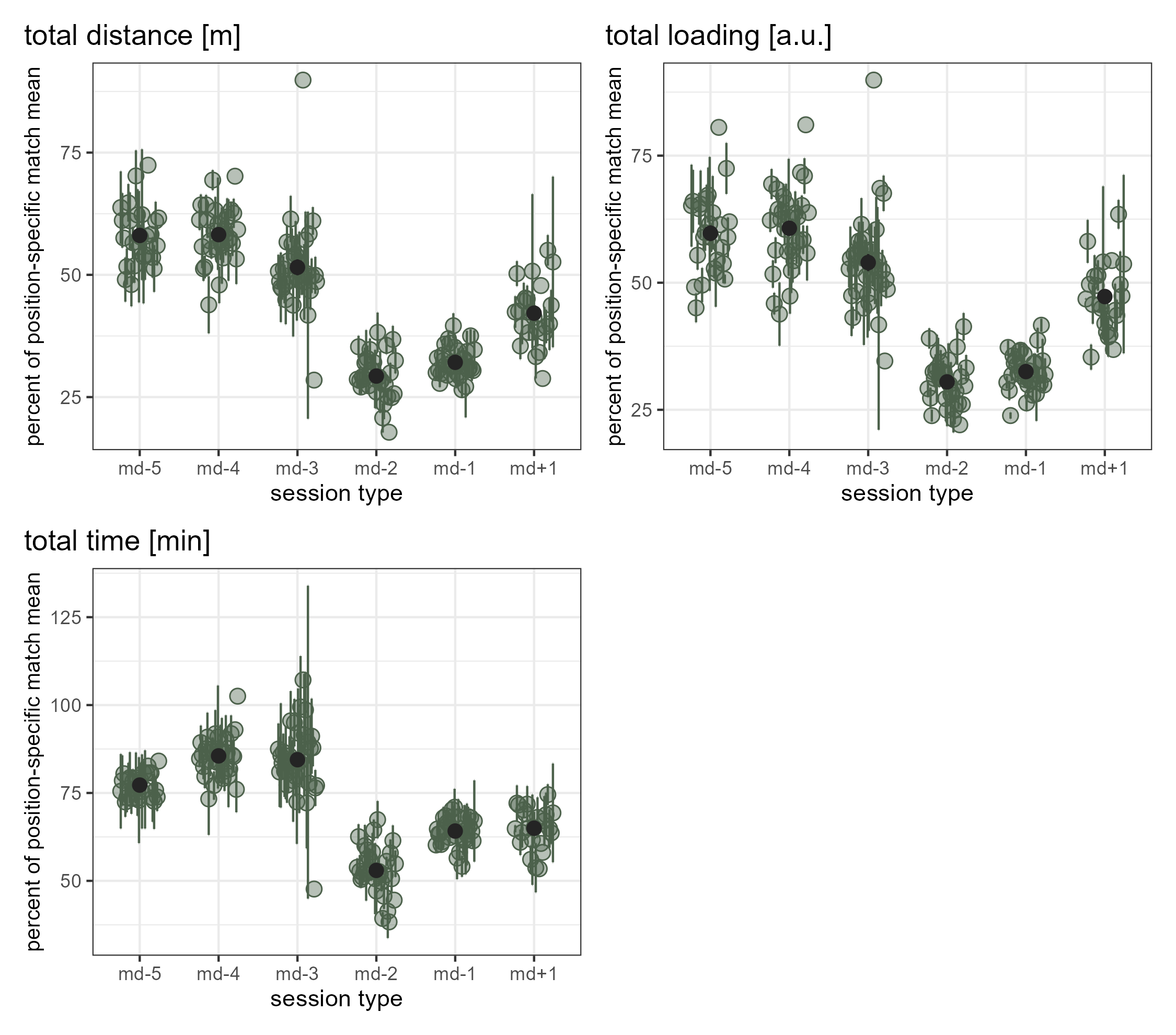

Supplement: Supplementary file 6 [file Image6.tiff]
